# Advancements in PSMA ligand radiolabeling for diagnosis and treatment of prostate cancer: a systematic review

**DOI:** 10.3389/fonc.2024.1373606

**Published:** 2024-03-21

**Authors:** Yuanzhuo Yan, Huixian Zhuo, Tengfei Li, Jintao Zhang, Min Tan, Yue Chen

**Affiliations:** ^1^ Department of Nuclear Medicine, The Affiliated Hospital of Southwest Medical University, Luzhou, Sichuan, China; ^2^ Nuclear Medicine and Molecular Imaging Key Laboratory of Sichuan Province, Luzhou, Sichuan, China; ^3^ Nuclear Medicine Institute of Southwest Medical University, Luzhou, Sichuan, China; ^4^ Department of Medical Imaging, Southwest Medical University, Luzhou, Sichuan, China

**Keywords:** PSMA, prostate cancer, mCRPC, radiolabeled, RLT, Tat, PET/CT

## Abstract

Prostate cancer(PCa), a leading global health concern, profoundly impacts millions of men worldwide. Progressing through two stages, it initially develops within the prostate and subsequently extends to vital organs such as lymph nodes, bones, lungs, and the liver. In the early phases, castration therapy is often employed to mitigate androgen effects. However, when prostate cancer becomes resistant to this treatment, alternative strategies become imperative. As diagnostic and treatment methodologies for prostate cancer continually advance, radioligand therapy (RLT) has emerged as a promising avenue, yielding noteworthy outcomes. The fundamental principle of RLT involves delivering radionuclide drugs to cancerous lesions through specific carriers or technologies. Subsequently, these radionuclide drugs release radioactive energy, facilitating the destruction of cancer cell tissues. At present, the positron emission tomography (PET) targeting PSMA has been widely developed for the use of diagnosis and staging of PCa. Notably, FDA-approved prostate-specific membrane antigen (PSMA) targeting agents, such as ^68^Ga-PSMA-11 and ^177^Lu-PSMA-617, represent significant milestones in enhancing diagnostic precision and therapeutic efficacy. This review emphasizes the current research status and outcomes of various radionuclide-labeled PSMA ligands. The objective is to provide valuable insights for the continued advancement of diagnostic and therapeutic approaches in the realm of prostate cancer.

## 1 Introduction

As of 2020, the global landscape of cancer incidence and mortality reveals a staggering burden, with around 19.3 million new cancer cases reported worldwide (excluding non-melanoma skin cancer). Unfortunately, approximately 10 million cancer patients succumbed to the disease (excluding non-melanoma skin cancer). Prostate cancer constituted a significant portion of these cases, with approximately 1.4 million new diagnoses globally, accounting for 7.3% of all cancer cases. Regrettably, there were approximately 375,000 deaths attributed to prostate cancer, making it the second most common cancer and the fifth leading cause of death among men in 2020 (1). The anticipated cancer burden in 2022 underscores the significant impact on public health in both China and the United States. It is projected that China will experience around 4.82 million new cancer cases and approximately 3.21 million cancer-related deaths. In China, prostate cancer specifically contributes over 125,000 new cases and more than 56,000 deaths. Similarly, the United States is expected to see around 2.37 million new cancer cases and approximately 640,000 cancer-related deaths in 2022. For prostate cancer in the United States, the estimates are approximately 126,900 new cases and 34,600 deaths. These figures highlight the substantial health challenges posed by cancer and the need for continued efforts in prevention, early detection, and effective treatment strategies (2). The data underscores a consistent rise in the incidence of prostate cancer, a trend attributed to advancements in medical technology. Improved diagnostic methods, heightened awareness prompting proactive screening, and demographic shifts, including an aging population, contribute to the increasing detection rates. As healthcare technologies continue to progress, early detection becomes more achievable, playing a pivotal role in effectively managing and treating prostate cancer (3, 4). While many prostate cancers exhibit slow growth, their impact on a patient’s health and life can be profound, potentially progressing into castration-resistant prostate cancer, which stands as the primary cause of death in prostate cancer patients. Hence, the early diagnosis and precise evaluation of prostate cancer hold immense significance in mitigating the potential adverse outcomes associated with the disease. Early detection allows for timely intervention and tailored treatment strategies, contributing to better patient outcomes and quality of life.

PET/CT stands as a widely utilized nuclear medicine technique for comprehensive tumor examinations. By integrating Positron Emission Tomography (PET) and Computed Tomography (CT), PET/CT provides valuable insights into the metabolic activity throughout the body. This imaging modality aids in the early detection of tumor lesions, offering crucial information on tumor size, location, local invasion, lymph node metastasis, and distant metastasis. Additionally, PET/CT serves as a valuable tool in distinguishing between focal changes and tumor recurrence following radiotherapy. Its versatility and effectiveness make PET/CT one of the most commonly employed methods for oncology evaluations today. Moreover, its application in targeted cancer therapy further enhances its value in contributing to improved patient care (5, 6).

^18^F-FDG (fluorodeoxyglucose) stands as a frequently employed PET tracer for assessing tumor metabolic activity and diagnosing tumor lesions. Its potential utility extends across various facets of prostate cancer care, encompassing diagnosis, staging, treatment evaluation, and prognosis, especially in cases of castration-resistant metastatic prostate cancer. The versatility of ^18^F-FDG PET proves valuable in providing comprehensive insights into prostate cancer, aiding in precise diagnosis, effective staging, informed treatment decisions, and prognostic assessments for patients (7, 8). While ^18^F-FDG is extensively utilized in oncology, it exhibits lower specificity, occasionally accumulating in inflammation, infections, and normal tissues. Additionally, it may be less sensitive to certain tumor types, such as breast and prostate cancer. In instances where these limitations are notable, alternative PET tracers may prove more suitable for achieving higher specificity and sensitivity in imaging and diagnosis (9–12). PSMA (Prostate Specific Membrane Antigen) is a membrane antigen characterized by high expression in prostate tissue and on the surface of prostate cancer cells. Due to its heightened expression in prostate cancer, PSMA has evolved into a pivotal marker for targeted diagnosis and treatment of prostate cancer. The application of PET-CT imaging with radionuclide-labeled PSMA has demonstrated significant potential in detecting and staging prostate cancer, offering a promising approach for improved visualization and assessment of the disease (13, 14). Currently, PSMA imaging has gained recognition in the latest international guidelines and is poised to become the forefront choice for the diagnosis and treatment of prostate cancer in the future. This acknowledgment underscores the increasing importance of PSMA-based imaging methods in refining the accuracy and precision of prostate cancer diagnostics, guiding targeted therapeutic approaches. As guidelines evolve, the prominence of PSMA imaging is expected to play a central role in the comprehensive management of prostate cancer (15). PSMA-617, PSMA-1007, PSMA-11, PSMA-I&T, and similar compounds are chemical reagents designed to target the prostate-specific membrane antigen (PSMA). They possess the ability to bind to PSMA present on the surface of tumor cells, facilitating the visualization of tumor signals and offering the potential for targeted therapy. These PSMA ligands are preferred due to their advantages, including small molecular weight, robust tissue permeability, rapid blood clearance, and ease of large-scale synthesis. Consequently, they have emerged as the primary choice for molecular imaging probes in prostate cancer, finding widespread applications in the targeted therapy of prostate cancer (13). This review seeks to provide a comprehensive overview of the current research status of commonly used PSMA ligand drugs for radioisotope labeling. The focus is on exploring their properties, efficacy, and toxicity, along with examining the outcomes of their combined application with other treatments. The goal is to offer a consolidated understanding of the current landscape of PSMA ligand drugs, shedding light on their characteristics, therapeutic effectiveness, and potential synergies when employed in conjunction with other therapeutic modalities.

## 2 Radionuclide label PSMA-ligand used for diagnosis

### 2.1 ^68^Ga-PSMA-11

^68^Ga-PSMA-11 is the small molecule imaging agent known for its favorable biological distribution characteristics. It was initially utilized in clinical settings in 2012, and by the end of December 2020, ^68^Ga-PSMA-11 had become the earliest PSMA imaging agent to receive approval from the FDA (U.S. Food and Drug Administration).

**Figure 1:**
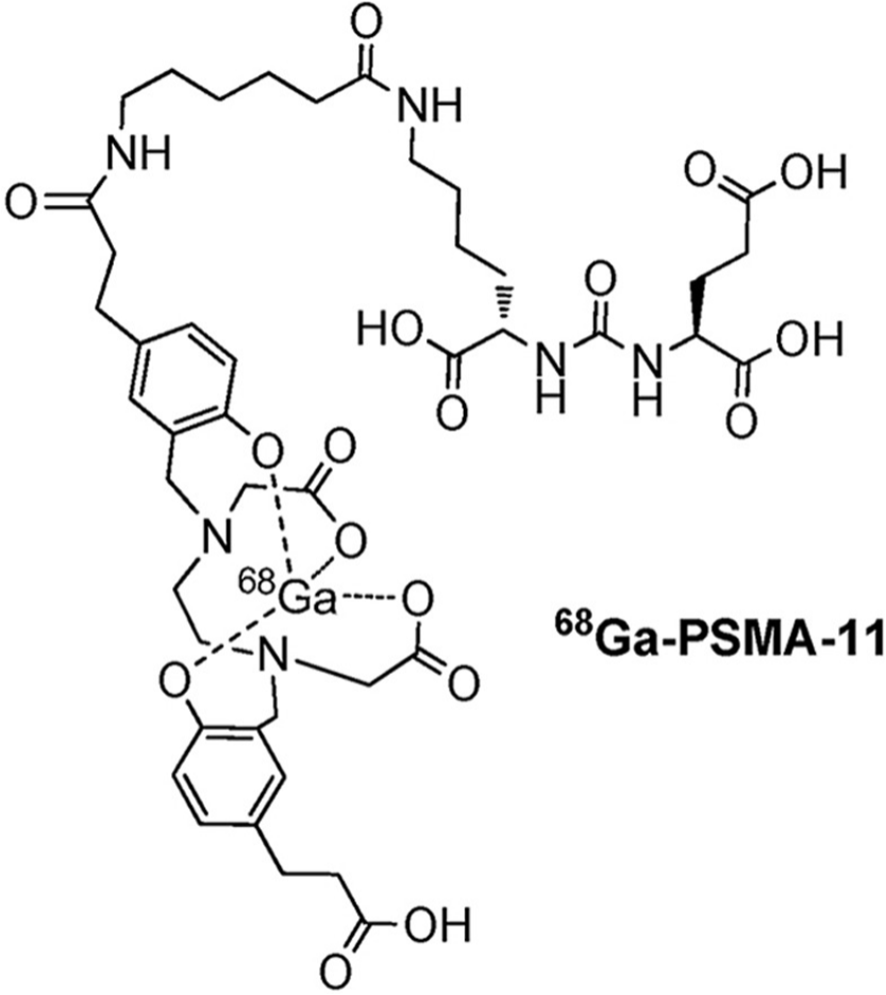
The chemical structure of ^68^Ga-PSMA-11.

The complexation of the radionuclide gallium-68 is achieved using the bifunctional non-cyclic chelating agent HBED-CC (N,N’-bis[2-hydroxy-5-(carboxyethyl)]-N,N’-diacetic acid). This chelating agent is hexadentate and adopts an octahedral geometry. In the complex, gallium-68 is coordinated with 2 nitrogen atoms, 2 hydroxyl groups, and 2 carboxyl groups. The ^68^Ga-HBED-CC group binds to the PSMA analogue Glu-NH-CO-NH-Lys(Ahx). **Figure 1** depicts the structure of ^68^Ga-PSMA-11 (16). ^68^Ga-PSMA-11 PET/CT has shown notable specificity and sensitivity in the diagnosis and staging of primary prostate cancer, re-staging of patients with recurrent prostate cancer (PCa), and evaluating castration-resistant prostate cancer (CRPC). The use of this imaging technique can contribute to more accurate diagnosis, staging, and assessment of treatment response in patients with prostate cancer.

^68^Ga-PSMA-11 PET/CT has shown notable specificity and sensitivity in the diagnosis and staging of primary prostate cancer, re-staging of patients with recurrent prostate cancer (PCa), and evaluating castration-resistant prostate cancer (CRPC). The use of this imaging technique can contribute to more accurate diagnosis, staging, and assessment of treatment response in patients with prostate cancer (17).

^68^Ga-PSMA-11 has been extensively demonstrated to exhibit high sensitivity and specificity in the diagnosis of prostate cancer. It significantly enhances the detection rate compared to other imaging agents such as ^18^F-FDG PET/CT, ^18^F-PSMA-1007 PET/CT, and ^64^CuCl_2_ PET-CT (18). In particular, ^68^Ga-PSMA-11 PET/CT displays higher sensitivity for prostate cancer diagnosis (19). Yang J. et al. utilized the ^68^Ga-PSMA-11 PET Maximum Normalized Threshold (SUVmax) to predict clinically significant prostate cancer (PCa) and PSA levels in the gray area (4-10 ng/ml), which is challenging for PCa diagnosis. In this study, the sensitivity and specificity were reported as 86.21% and 86.54%, respectively. ^68^Ga-PSMA-11 PET facilitates the screening and early diagnosis of prostate cancer and can help avoid unnecessary biopsy procedures (20).

In a study conducted by Hope et al., ^68^Ga-PSMA-11 PET imaging scans were employed for preliminary staging in 764 patients with prostate cancer and pelvic lymph node metastasis. The results indicated a positive ^68^Ga-PSMA-11 PET scan, with reported sensitivity and specificity for pelvic lymph node metastasis of 0.40 and 0.95, respectively (21). Consequently, ^68^Ga-PSMA-11 PET is considered beneficial for preoperative staging and assisting in lymph node dissection. The study findings suggest that ^18^F-PSMA-11 PET/MRI can help reduce false negatives for clinically significant prostate cancer (csPCa) when compared to MRI alone. This potential improvement in diagnostic accuracy may lead to a reduction in the number of unnecessary prostate biopsies required to diagnose clinically significant prostate cancer. The combined information from PET and MRI imaging could enhance the detection and localization of prostate cancer, thereby aiding in more targeted and effective clinical decision-making (22).

Indeed, ^68^Ga-PSMA-11 PET is increasingly recommended by various guidelines for detecting biochemical recurrent prostate cancer. Its high accuracy in detection, along with its capability to assess stage and prognosis, has contributed to its recognition and adoption in clinical practice. This imaging modality has proven valuable in the management of patients with biochemical recurrence by providing detailed information about the location and extent of disease, aiding in treatment planning, and contributing to more informed clinical decision-making (23, 24). This study examining 635 cases involving prostatectomy and/or radiotherapy with ^68^Ga-PSMA-11 PET scans and histopathological verification, the researchers found that the detection rates varied with different PSA levels. The results were as follows: for PSA levels <0.5ng/mL (n=136), the detection rate was 38%; for PSA levels 0.5 to <1.0ng/mL (n=79), the detection rate was 57%; for PSA levels 1.0 to <2.0ng/mL (n=89), the detection rate was 84%; for PSA levels 2.0 to <5.0ng/mL (n=158), the detection rate was 86%. The detection rate increased to 97% for PSA levels ≥5.0ng/mL (n=173, P<.001). The study concluded that the rate of PSA detection in localized recurrent prostate cancer with ^68^Ga-PSMA-11 PET was significantly improved, demonstrating the effectiveness of this imaging modality in detecting recurrent disease at various PSA levels (23). The multicenter study involving 138 prostate cancer (PCa) patients with biochemical recurrent (BCR) lesions, classified as progressive, mixed, or nonprogressive, utilized quantitative parameters (SUVmean, SUVmax, SUVpeak, volume) to quantify tumor response at a focal level following ^68^Ga-PSMA-11 PET scans. The study demonstrated that patients with systemic progression had a significantly higher risk of death compared to those without progression (HR=5.70), with SUVmean identified as having the highest prognostic value. Furthermore, ^68^Ga-PSMA-11 PET was found to possess significant prognostic value in progressive patients for overall survival (HR=3.67). These findings suggest that ^68^Ga-PSMA-11 PET can assist in restaging biochemical recurrent prostate cancer and plays a crucial role in assessing prognosis (24).

^68^Ga-PSMA-11 PET/CT stands out as a valuable tool for evaluating metastatic castration-resistant prostate cancer (mCRPC), enhancing the precision of staging, and aiding in crucial clinical decision-making processes. With its high sensitivity and specificity, this imaging modality provides detailed insights into the localization and extent of metastatic lesions, empowering doctors to make well-informed decisions on treatment strategies and patient management for those facing advanced prostate cancer (25, 26). In contrast to other imaging techniques, ^68^Ga-PSMA-11 PET proves highly effective in detecting small, distant, and atypical metastases associated with prostate cancer, including instances of intraocular (27) and isolated peritoneal metastases (28).

### 2.2 ^18^F-PSMA-1007

**Figure 2:**
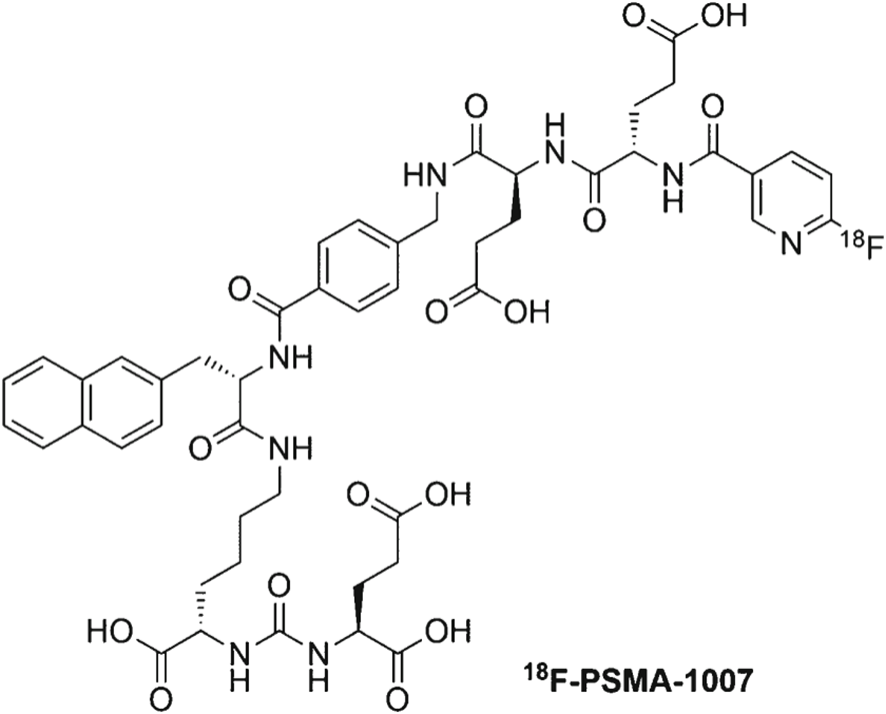
The chemical structure of ^18^F-PSMA-1007.

PSMA-1007 represents a novel PSMA ligand derived from the chemical structure of PSMA-617 (**Figure 2**). It targets the gluu-urea-lys motif of the PSMA enzyme pocket S1` and concurrently engages the naphthaln-based hydrophobic accessory pocket S1. The primary distinction lies in the addition of two glutamic acids to the site carrying the radioactive label, simulating the carboxyl group of the DOTA chelating agent (29). Prostate-specific membrane antigen (PSMA) is found in the proximal tubule cells of the kidney, resulting in significant uptake of renal tracers in PSMA-PET. This uptake may contain valuable information about renal function. PSMA-1007 has been validated for its excellent binding and internalization properties *in vitro*. It exhibits high specific uptake *in vivo* and demonstrates effective differentiation between normal ganglia and lymph node metastasis in prostate cancer. Research indicates that PET/CT diagnosis using ^18^F-PSMA-1007 offers advantages over traditional imaging modalities such as CT, MRI, ultrasound, and bone imaging (30). Moreover, in contrast to other well-known PSMA ligands, PSMA-1007 primarily undergoes metabolization through the hepatobiliary pathway, diverging from the renal metabolism observed with other ligands. This distinctive metabolic pathway could potentially confer benefits in the differentiation of lymph node metastasis, particularly in patients with recurrent prostate cancer (31, 32). In a retrospective survey involving 73 prostate cancer patients, Rassek P et al. concluded that renal uptake of ^18^F-PSMA-1007 can serve as an accurate measure for quantifying renal function, utilizing parameters such as SRFPSMA-TOTAL or SRFSUV (33).. In the latest clinical study, which included 60 patients diagnosed with prostate cancer exhibiting low PSA levels, ^18^F-PSMA-1007 PET/MRI detected 53 lesions in 45 patients, resulting in a detection rate of 75%. The average PSA value in this cohort was 0.31 ng/mL. These findings suggest that ^18^F-PSMA-1007 proves to be an excellent molecular probe, particularly beneficial for early-stage biochemical recurrence (BCR) patients with exceptionally low PSA levels (34). Given the chemical structure and biological activity, ^18^F-PSMA-1007, along with ^177^Lu-PSMA-617, holds promise in matching the diagnostic capabilities. Further research endeavors will ascertain the clinical significance of this ligand and its potential for practical application in clinical settings. Ongoing studies will shed light on the utility and effectiveness of this ligand, shaping its role in clinical diagnosis and potentially opening avenues for enhanced prostate cancer management (29, 35, 36).

### 2.3 18F-DCFPyL

**Figure 3:**
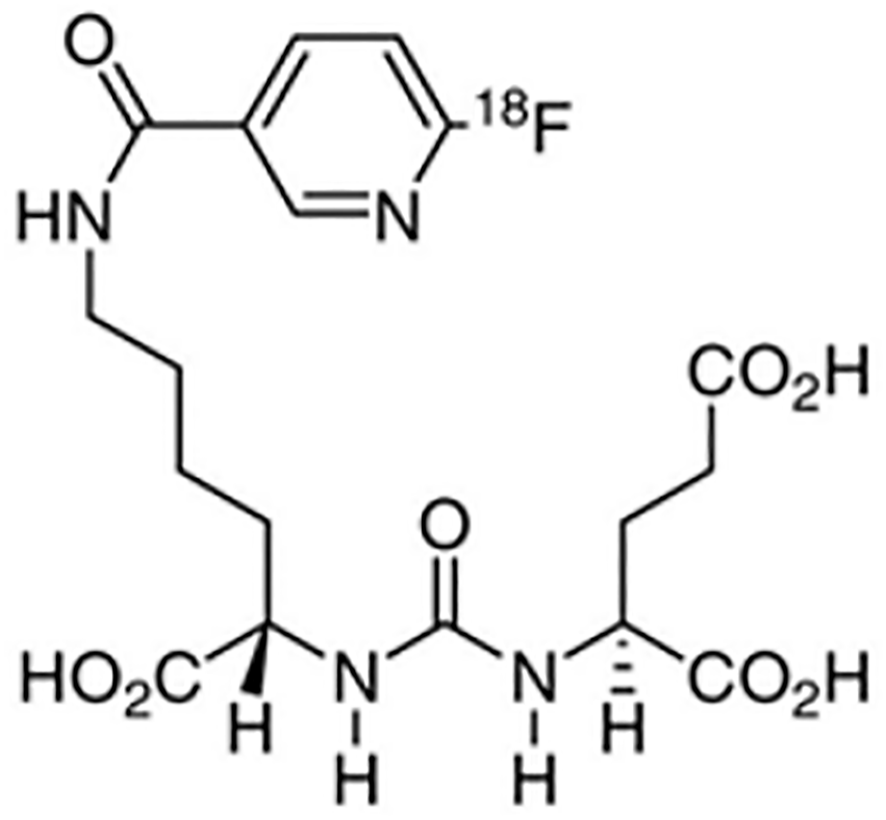
The chemical structure of ^18^F-DCFPyL.

^18^F-DCFPyL, or 2-(3-(1-carboxy-5-[(6-[^18^F]fluoro-pyridine3-carbonyl)-amino]-pentyl)-ureido)-pentanedioic acid, is a specific small molecule imaging agent developed based on the Glu-urea-Lys structure (**Figure 3**). This agent demonstrates high affinity for binding to the extracellular region of Prostate-Specific Membrane Antigen (PSMA) (37). It is also the second FDA-approved PSMA-targeted PET imaging drug (38).

^18^F-DCFPyL has been validated in multiple prospective clinical trials such as OSPREY and CONDOR, demonstrating its effectiveness in clinical applications, including staging, re-staging, and efficacy evaluation in patients with prostate cancer (PCa). In the Phase III multicenter CONDOR trial, ^18^F-DCFPyL-PET/CT imaging was conducted on 208 patients with prostate adenocarcinoma who had undergone radical prostatectomy (RP) due to biochemical recurrence and had negative results on standard imaging. The administered dose was 333 MBq, and it was given intravenously 1 to 2 hours before PET/CT imaging. The results revealed that 63.9% of the patients altered their intended treatment plan after ^18^F-DCFPyL-PET/CT, and the disease detection rate ranged from 59% to 66% (39). Hence, ^18^F-DCFPyL-PET/CT proves to be an effective tool for disease imaging in patients with recurrent prostate cancer. In the OSPREY trial, ^18^F-DCFPyL-PET/CT examinations were conducted on 252 male patients with high-risk prostate cancer after radical prostatectomy plus pelvic lymph node dissection. The median specificity and sensitivity were reported as 97.9% (95% CI: 94.5% to 99.4%) and 40.3% (95% CI: 28.1% to 52.5%), respectively (40), The collective evidence supports the utility of ^18^F-DCFPyL-PET/CT for detecting lesions in patients with prostate cancer (PCa) and evaluating the staging of lymph node or distant metastasis. Zsolt Szabo was the first to employ ^18^F-DCFPyL in patients with hormone-independent or castration-resistant prostate cancer. Studies have demonstrated that ^18^F-DCFPyL exhibits physiological uptake in salivary glands, lacrimal glands, kidneys, liver, spleen, and small intestine, with no uptake observed in the brain. Simultaneously, it is excreted through urine, showing notable accumulation in the kidneys and bladder. Dosimetry studies revealed that the effective dose of ^18^F-DCFPyL was 0.0165 mSv/MBq or 6.1 mGy (0.61 rem) at an injected dose of 370 MBq. There was significant accumulation in prostate cancer foci (SUVmax up to 9100, tumor/blood ratio up to 950). The highest radiation doses were observed in renal viscera (0.0945 mGy/MBq), bladder wall (0.0864 mGy/MBq), submandibular gland (0.0387 mGy/MBq), and liver viscera (0.0380 mGy/MBq) (41).

Despite the relatively short duration of clinical application, ^18^F-DCFPyL has undergone several evaluations, all of which report high sensitivity, specificity, and positive detection rates. In a prospective study involving 205 patients experiencing biochemical recurrence (BCR) after initial radical prostate cancer surgery or radiation therapy, separate examinations were conducted using ^18^F-DCFPyL PET/CT and ^18^F-fluoromyclocholine PET/CT. The positive detection rate of lesions increased with the rise in PSA value. The overall detection rate of ^18^F-DCFPyL PET/CT was superior to that of 18F-fluoromethylcholine PET/CT (58% vs 40%, p < 0.0001) (42). The ^18^F-PSMA PET/CT pair can also be utilized to detect uncommon metastatic sites, including the brain, liver, and penis, among others (43). Simultaneously, when compared to other traditional imaging modalities, ^18^F-DCFPyL PET/MR demonstrates superiority in pre-treatment screening of prostate cancer patients with pretreatment lesion localization. In a study led by Adriano Basso Dias et al., ^18^F-DCFPyL PET/MRI was employed to screen prostate cancer patients undergoing focal ablation therapy (FT). mpMRI and/or PET/MRI were conducted on 34 patients with low/medium-risk PCa. ^18^F-DCFPyL PET/MRI excluded focal treatment in nearly 30% of patients with low/medium-risk PCa and exhibited higher sensitivity (97% vs 76%, P = 0.02) but relatively lower specificity (30% vs 85%, P < 0.001). As a result, its elevated sensitivity effectively detects lesions without missing detections, enhancing the diagnostic efficacy of clinically significant (CS) prostate cancer in patients undergoing focal ablation (FT). Nonetheless, its lower specificity may restrict the use of PET/MRI as a screening tool (44). Therefore, in conjunction with histological characteristics and conventional imaging examinations, ^18^F-PSMA PET/CT can be more effectively applied in patients with prostate cancer. NCT04461509 is a Phase II clinical trial, which is utilizing ^18^F-PSMA PET/MRI and enhanced prostate imaging with standard mp/MRI to evaluate the effectiveness of focal high-intensity focused ultrasound (HIFU) therapy on prostate cancer targets Currently. ^18^F-DCF PET/CT imaging facilitates individualized management of prostate cancer by eliminating unnecessary biopsies through disease staging and risk stratification (39, 45).

The ^18^F labeled PSMA targeting compound provides a significant improvement in image quality and noise compared to the ^68^Ga-labeled PSMA-targeting compound. This improvement allows for the detection of subtle lesions, and the longer half-life of 110 minutes facilitates delayed imaging (46).

## 3 Radionuclide label PSMA-ligand used for therapy

### 3.1 ^177^Lu-PSMA-617

Although many treatments have emerged for metastatic castration-resistant prostate cancer (mCRPC) over the past decades, recent clinical trials have shown a survival benefit of ^177^Lu-PSMA-617 in mCRPC following chemotherapy (47). Currently, a relatively novel treatment method for metastatic castration-resistant prostate cancer (mCRPC) involves targeted radioactive oligonucleotide therapy. In this approach, radioactive isotopes are paired with monoclonal antibodies targeting cancer-specific antigens, such as prostate-specific membrane antigen (PSMA). This method is not only simple but also minimizes the impact on normal tissues.

**Figure 4:**
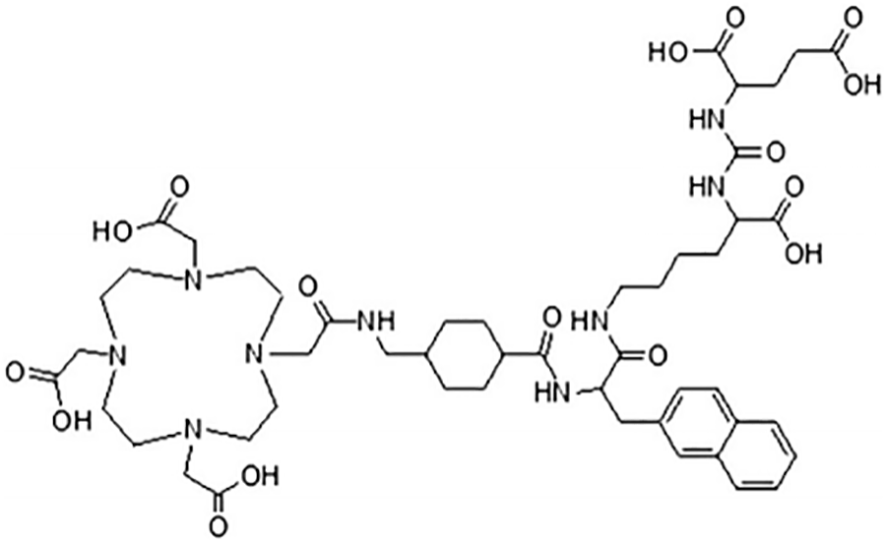
The chemical structure of PSMA-617.

PSMA-617, a novel DOTA-conjugated PSMA inhibitor containing naphthalene, displays elevated uptake in both tumors and kidneys within the LNcaP tumor model, as observed through small animal PET imaging. It efficiently internalizes into LNcaP cells and exhibits swift renal clearance, making it promising for therapeutic applications. The favorable pharmacokinetics lead to target-to-non-target ratios of 1058 (tumor-to-blood) and 529 (tumor-to-muscle) at 24 hours post-injection in animal models (48). This ligand is amenable to labeling with ^68^Ga, ^111^In, ^177^Lu, and ^90^Y (**Figure 4**). Early clinical studies have showcased the high-contrast detection capabilities of ^68^Ga-PSMA-617 in identifying prostate cancer (PCa) lesions. Moreover, it has been utilized in the treatment of metastatic PCa across various medical centers (49, 50).

There is a substantial body of randomized clinical evidence and practical experience regarding the use of the PSMA radioligand ^177^Lu-PSMA-617 for therapeutic purposes. Since obtaining regulatory approval in 2013, Germany and several other regions in Europe have accumulated significant experience in the application of ^177^Lu-PSMA-617 for prostate cancer treatment (51–53). In a randomized, parallel-group, open-label, phase 2 non-inferiority trial, Swayamjeet Satapathy et al. prospectively compared the efficacy and safety of ^177^Lu-PSMA-617 with docetaxel in patients with metastatic castration-resistant prostate cancer (mCRPC). A total of 40 patients underwent randomization, and the best prostate-specific antigen response rate (PSA-RR) was 60% (9/15) in the 177Lu-PSMA-617 group and 40% (8/20) in the docetaxel group. The difference in PSA-RR between the two groups was 20% (95% confidence interval, CI: -12-47, P=0.25). Furthermore, the 6-month progression-free survival rates for the ^177^Lu-PSMA-617 group and the docetaxel group were 30% and 20%, respectively (difference 10%, 95% CI: -18-38, P=0.50). The study concludes that ^177^Lu-PSMA-617 is safe and non-inferior to docetaxel in the treatment of mCRPC and can be considered during early stages of the disease process (54). As of March 2022, the FDA has granted approval for the use of the drug exclusively in the treatment of patients diagnosed with PSMA-positive metastatic castration-resistant prostate cancer. This approval is specifically designated for individuals who have undergone previous treatments, such as androgen receptor inhibition or taxane chemotherapy (55). In a phase III trial conducted by O. Sartor et al., 831 out of 1179 screened patients were enrolled for randomization between June 2018 and mid-October 2019. The findings revealed that ^177^Lu-PSMA-617 led to a significant extension in progression-free survival compared to standard therapy. Despite a higher incidence of grade 3 or higher adverse events in patients using ^177^Lu-PSMA-617 compared to those who did not, their overall quality of life was not significantly impacted (56).

The fundamental principle of radioactive isotope therapy involves delivering a high dose of radiation to target tissues while minimizing toxicity to healthy tissues. The prerequisite for treatment is the presence of a PSMA-positive tumor phenotype detected through PET or imaging. The ^177^Lu-PSMA-617 treatment appears to be a safe approach for castration-resistant prostate cancer. The maximum tolerated dose for a single administration may range between 7.4-11.1 GBq, depending on whether the tumor involves the bone marrow (57). Early inclusion of clinical patients in studies has shown that the distribution of lesions and physiological uptake regions is similar to what is observed in early diagnostic ^68^Ga-PSMA-11 PET scans. This similarity indicates promising therapeutic potential for ^177^Lu-PSMA-617 treatment in castration-resistant prostate cancer (58).

### 3.2 ^177^Lu-PSMA- I&T

**Figure 5:**
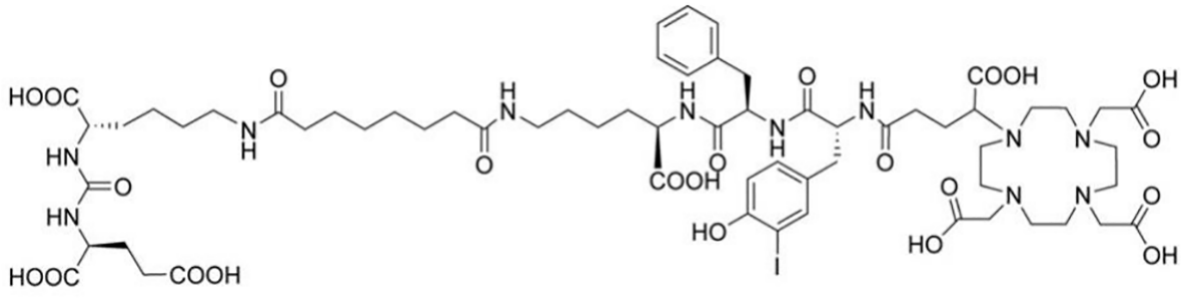
The chemical structure of PSMA-I&T.

PSMA I&T, or DOTAGA-(I - y) fk-fk (Sub - KuE), with DOTAGA [1,4,7,10-tetraazacyclododececane-1(-glutaric acid)-4,7,10-triacetic acid, 1,4,7, 10-tetraazecyclodecadecane-1-(glutamic acid)-4,7,10-triethonic acid] as a chelating agent (59, 60). This small molecule PSMA-targeted inhibitor exhibits rapid pharmacokinetics and high affinity for PSMA (**Figure 5**). PSMA I&T can be labeled with isotopes such as ^68^Ga, ^111^In, ^177^Lu, and ^225^Ac for imaging and therapeutic purposes (61).

Compared to ^177^Lu-PSMA-617, ^177^Lu-PSMA-I&T demonstrates similar mean tumor dose absorption and favorable safety profiles. Notably, ^177^Lu-PSMA-I&T exhibits lower uptake in salivary glands, resulting in reduced potential damage to these glands. However, it is important to consider that the kidney uptake rate is relatively high with ^177^Lu-PSMA-I&T (54, 62, 63). ^177^Lu-PSMA-I&T is utilized for targeted radionuclide therapy and has shown promising efficacy in patients with metastatic castration-resistant prostate cancer (mCRPC). Several clinical trials are currently underway, including NCT05204927, NCT05867615, and NCT04647526. NCT05204927 is a prospective, multicenter, randomized Phase 3 clinical trial involving 400 metastatic prostate cancer patients randomized to receive either ^177^Lu-PSMA-I&T or hormone therapy. The trial aims to assess disease progression based on solid tumor response criteria and record PSA levels and symptoms.In a retrospective study conducted by Amir Karimzadeh et al., 301 mCRPC patients treated with ^177^Lu-PSMA-I&T were evaluated. The standard activity of ^177^Lu-PSMA-I&T was 7.4 GBq, administered every 4-10 weeks (median, 6 weeks) for a total of 1138 cycles of intravenous injection (median, three cycles per patient). Results indicated that 34% of patients demonstrated at least a 50% PSA response, with a median progression-free PSA survival of 16.0 weeks and an overall survival (OS) of 13.8 months (64). Mehmet Onur Demirkol et al. conducted ^177^Lu-PSMA-I&T radionuclide therapy (RLT) on 33 patients with metastatic castration-resistant prostate cancer (mCRPC) and 5 patients with metastatic hormone-sensitive prostate cancer (mHSPC). Among the mCRPC patients, 56% exhibited a PSA response of ≥30%. Notably, all mHSPC patients showed a high PSA response ranging from 93.0% to 99.9%. These findings suggest that ^177^Lu-PSMA-I&T RLT demonstrates significant antitumor activity. However, it is important to note that some patients experienced mild renal impairment or anemia during the course of treatment (65).

Furthermore, ^68^Ga-PSMA-I&T PET/CT has demonstrated successful utility in detecting primary lesions and staging prostate cancer patients. This approach offers advantages in the stratification and follow-up of patients undergoing treatment with the 177Lu-PSMA-I&T (Integrated Diagnosis and Treatment) radioligand (60, 66). ^225^Ac-PSMA-I&T has demonstrated enhanced anti-tumor effects in patients with advanced metastatic castration-resistant prostate cancer (mCRPC) (67). A Phase I/II clinical trial named AlphaBet (NCT05383079) is underway, combining ^223^Ra with ^177^Lu-PSMA-I&T. This combination aims to improve outcomes for patients with mCRPC and bone metastases (68).

### 3.3 ^225^Ac -PSMA-617

Alpha-targeted therapy (TAT) is a therapeutic approach that targets cancer cell vectors based on drugs labeled with radionuclides that emit alpha particles (69). Alpha-nuclides possess distinctive characteristics, including high linear energy transfer, a limited range, and potent cytotoxicity. The current alpha-radionuclides deemed suitable for targeted therapy encompass ^149^Tb, ^212/213^Bi, ^212^Pb (^212^Bi), ^225^Ac, and ^226/227^Th. These radionuclides show promise in targeted alpha-particle therapy due to their ability to deliver focused and intense radiation, making them valuable candidates for precision cancer treatment (70). Due to its extended half-life (t_1/2 = _10 days), distinctive decay properties, ease of coordination, and selective destruction of cancer cells with minimal damage to normal tissue, ^225^Ac stands out as one of the most favorable choices for Targeted Alpha Therapy (TAT). The unique characteristics of ^225^Ac make it an ideal candidate for precision cancer treatment, offering the potential to effectively combat cancer while minimizing harm to surrounding healthy tissues (71). Research findings indicate that ^225^Ac-PSMA-617 has demonstrated efficacy in patients with metastatic castration-resistant prostate cancer (mCRPC). In a groundbreaking study conducted by Clemens Kratochwil et al. in 2016, they pioneered the use of an alpha nuclide-labeled PSMA ligand for human therapy. Two patients in the study received treatment with ^225^Ac-PSMA-617 Radionuclide Therapy (RLT) at a dosage of 100 kBq/kg of body weight every 2 months. Remarkably, these patients exhibited a significant reduction in prostate-specific antigen (PSA) levels, suggesting the therapeutic potential of ^225^Ac-PSMA-617 in managing mCRPC (72).

The evaluation of ^225^Ac-PSMA therapy is currently limited by the scarcity of prospective, randomized trials, with ongoing trials such as NCT04597411 aiming to provide more robust insights. In a recent multi-center retrospective study, 488 patients with metastatic castration-resistant prostate cancer (mCRPC) underwent treatment with ^225^Ac-PSMA for a total of 1174 cycles (median 2 cycles, IQR 2-4). The patients were followed for a median duration of 9 months (IQR 5-17.5). The study reported a median overall survival of 15.5 months (95% CI 13.4 to 18.3) and a median progression-free survival of 7.9 months (6.8 to 8.9). These findings contribute to the evolving understanding of the efficacy and outcomes associated with ^225^Ac-PSMA therapy in mCRPC patients (73). Madhav Prasad Yadav et al. conducted a study involving 28 patients with metastatic castration-resistant prostate cancer (mCRPC) who were treated with ^225^Ac-PSMA-617. The average administered activity was 26.5 ± 12 MBq (range, 9.25-62.9 MBq), with a median of 3 cycles (range, 1-7 cycles). Following the first cycle and at the 8th week, PSA reduction of >50% was observed in 25% and 39% of patients, respectively. The median progression-free survival (PFS) and overall survival (OS) were reported as 12 months (95% CI: 9-13 months) and 17 months (95% CI: 16 months - not reaching the upper limit), respectively. The disease control rates were 82% and 63.6%, contributing valuable insights into the clinical outcomes of ^225^Ac-PSMA-617 therapy in mCRPC patients (74). Therefore, ^255^Ac-PSMA-617 RLT not only has good anti-tumor effect, but also has good therapeutic safety.

Moreover, the unique capability of alpha rays to eliminate cells that typically display resistance to beta or gamma rays, as well as chemotherapy drugs, positions ^225^Ac-PSMA-617 treatment as a compelling alternative. This makes it a viable option for patients facing tumors that have developed resistance to the conventional treatment with ^177^Lu-PSMA-617. The distinctive attributes of alpha radiation introduce a promising avenue for therapeutic intervention, particularly in addressing cells resistant to established treatment approaches (71). Nalan Alan-Selcuk et al. administered ^225^Ac-PSMA-617 to 23 mCRPC patients who had previously undergone unsuccessful ^177^Lu-PSMA-617 treatment (2-9 cycles). The median interval between doses was 13 weeks (range, 8-28 weeks), with an average dose activity of 7.6 MBq (range, 6.2-10.0 MBq) per cycle. Following the first treatment cycle (n=18), 50% of patients (n=9) exhibited disease control based on prostate-specific membrane antigen PET progression criteria. The median progression-free survival was 3.1 months, and the median overall survival was 7.7 months (75).

The most prevalent adverse effect of ^225^Ac-PSMA-617 was dry mouth (72–74, 76–78), and when administered in conjunction with ^177^Lu-PSMA-617, it may lead to a substantial rise in salivary toxicity. Delayed nephrotoxicity has also been documented following 2 cycles of ^225^Ac-PSMA-617 RLT (79).

## 4 Discussion

The article comprehensively examines the role of diverse radiolabeled PSMAs in both diagnosing and treating prostate cancer. It aims to consolidate the clinical evidence supporting various PSMA ligands in the context of prostate cancer. The degree of PSMA uptake serves as a crucial biomarker for prostate cancer, with elevated PSMA levels typically signifying the presence of prostate cancer cells in specific regions. Active cancer cells often exhibit heightened PSMA uptake, making PSMA a valuable indicator for prostate cancer, metastasis, or lymph node involvement. This information plays a pivotal role in guiding treatment decisions for prostate cancer patients. Additionally, for individuals undergoing PSMA radionuclide therapy, alterations in PSMA uptake levels can be utilized to assess the treatment’s effectiveness, where a reduction in uptake may indicate a positive treatment response on the tumor (25). Earlier investigations have established that PSMA (Prostate-Specific Membrane Antigen) is a membrane protein that exhibits high expression in prostate tissue. Normally, PSMA is predominantly present on the luminal surface of prostate epithelial cells. However, in cases of prostate cancer, the expression of PSMA significantly elevates, resulting in cancer cells displaying a heightened affinity for PSMA. This characteristic allows tracers like ^68^Ga-PSMA to selectively accumulate *in vivo* within prostate cancer cells that overexpress PSMA, as detected by positron emission tomography (PET-CT). Importantly, there is less accumulation of these tracers in normal tissues (23, 80).

While PSMA serves as a valuable visualization tool, it comes with certain limitations in comparison to the widely used imaging agent ^18^F-FDG. One notable limitation is the high uptake of PSMA in the liver, kidneys, and salivary glands. This heightened uptake in these normal tissues may potentially impact the diagnostic efficacy of PSMA imaging (31). The strength of PSMA lies in its comprehensive role in both radiological diagnosis and treatment. Building on the foundation of ^68^Ga-PSMA-11 imaging diagnosis, the administration of ^177^Lu-PSMA-617 for treatment is a significant advancement. Following treatment and the assessment of recurrent lesions in the early stages, ^68^Ga-PSMA-11 continues to exhibit promising effects. Leveraging the radioactivity of ^177^Lu, ^177^Lu-PSMA-617 serves as a targeted therapeutic agent for prostate cancer. It releases beta rays within the body, facilitating the localized destruction of cancer cells. With its high affinity for PSMA, it selectively targets tumor cells, demonstrating efficacy in the local treatment of refractory prostate cancer and metastatic disease, forming an integral part of various treatment regimens (81). Furthermore, ^225^Ac-PSMA-617 holds particular significance for patients exhibiting resistance to ^177^Lu-PSMA-617. The unique properties of ^225^Ac nuclide, including high energy, short range, potent cytotoxicity, and easy coordination, have elevated it to a recent hotspot in the realm of radioligand therapy (RLT) for patients with metastatic castration-resistant prostate cancer (mCRPC) (82). However, a common adverse reaction post-treatment is dry mouth. Concomitant administration with ^177^Lu-PSMA-617 may result in a notable escalation in salivary toxicity. Simultaneously, careful attention must be directed towards nephrotoxicity that may arise subsequent to the treatment (78).

As a diagnostic and therapeutic tool, the substantial uptake of PSMA in the salivary glands and urinary tract necessitates modification to enhance its diagnostic and therapeutic efficacy. Addressing this issue is crucial in future PSMA research. Moreover, future studies should explore additional structural variants of PSMA to ameliorate certain challenges associated with existing PSMA ligand structures.

## 5 Conclusion

**Figure 6:**
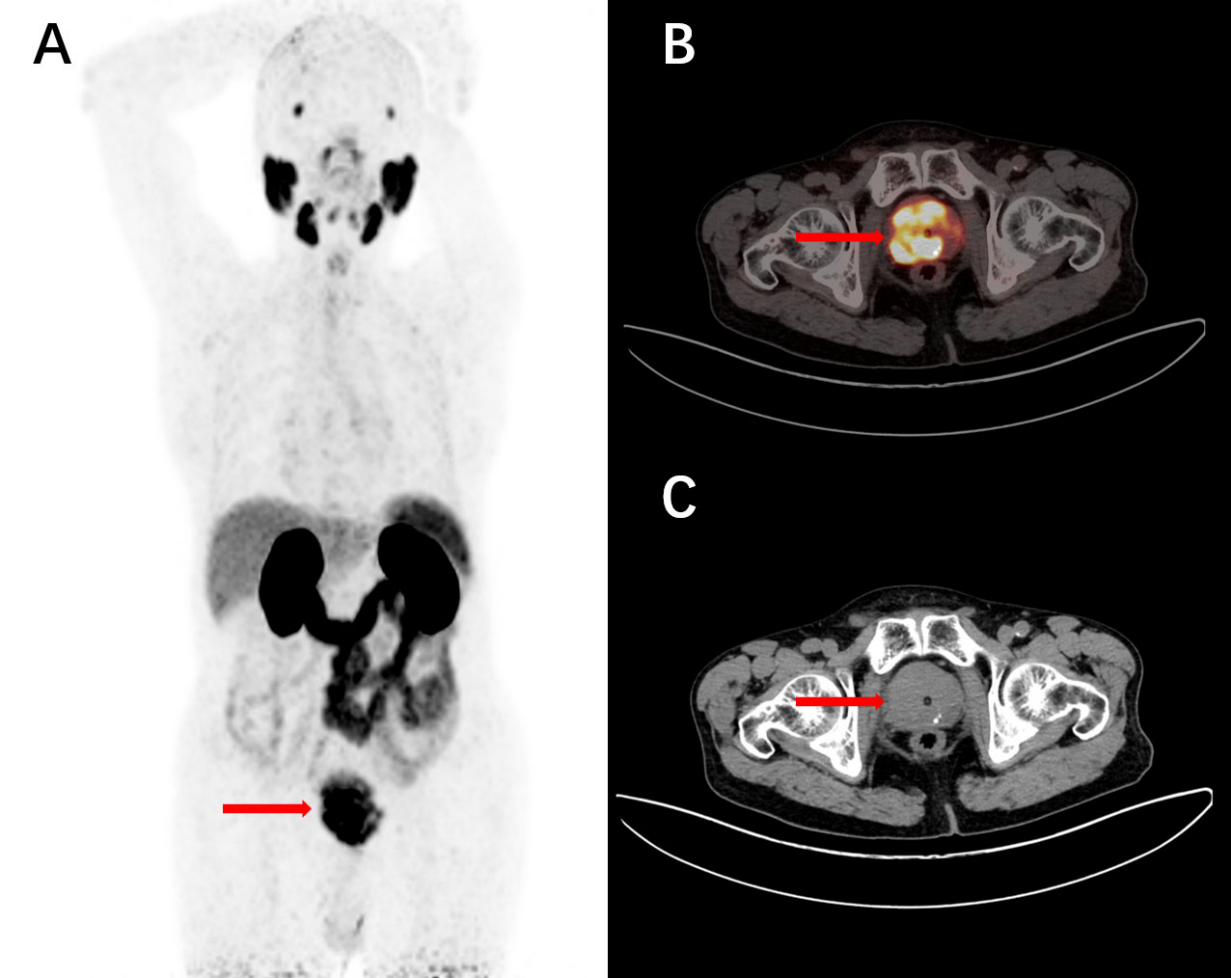
A 77-year-old male, diagnosed with prostate acinar adenocarcinoma through a biopsy, underwent intravenous injection of ^68^Ga-PSMA-11. 1 hour later, a PET/CT scan was performed, and the maximum intensity projection (MIP) image showed unevenly increased PSMA expression **(A)**. The tomographic images revealed non-uniform internal density, small nodular low-density shadows, and scattered calcifications with increased tracer uptake **(B, C)**. These findings are consistent with the presentation of prostate cancer.

**Figure 7:**
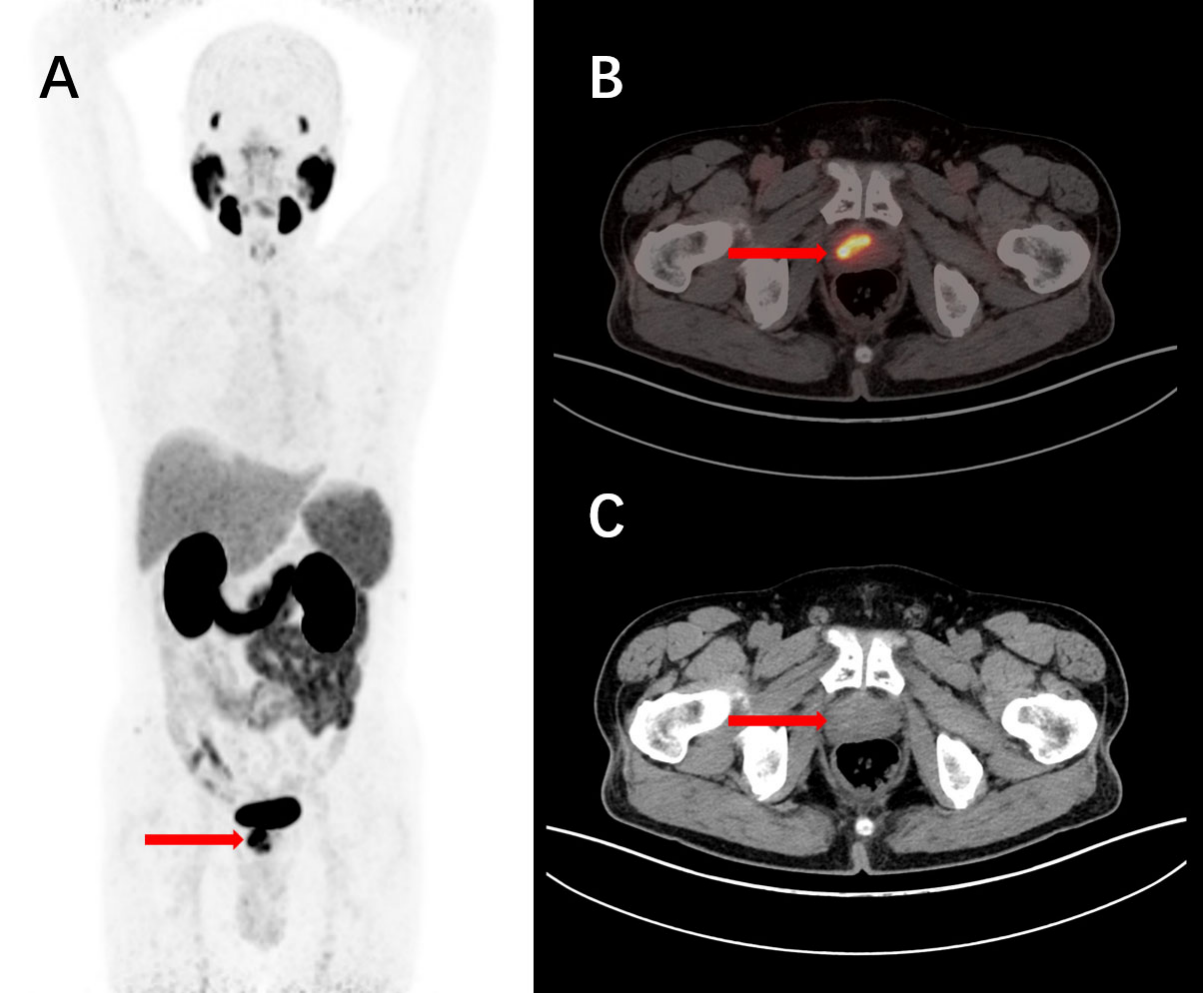
A 76-year-old male, diagnosed with prostate cancer for over 4 years and untreated, underwent intravenous injection of ^68^Ga-PSMA-11 followed by PET/CT tomographic imaging. The maximum intensity projection (MIP) image showed an elevated focal uptake in the nodular structure of the prostate **(A)**. The tomographic images revealed a nodular increased PSMA expression focus in the right portion of the prostate parenchyma, with a slightly decreased density in the corresponding area. Nodular high-density shadows were observed within the parenchyma **(B, C)**. These findings are consistent with the manifestation of prostate cancer.

**Figure 8:**
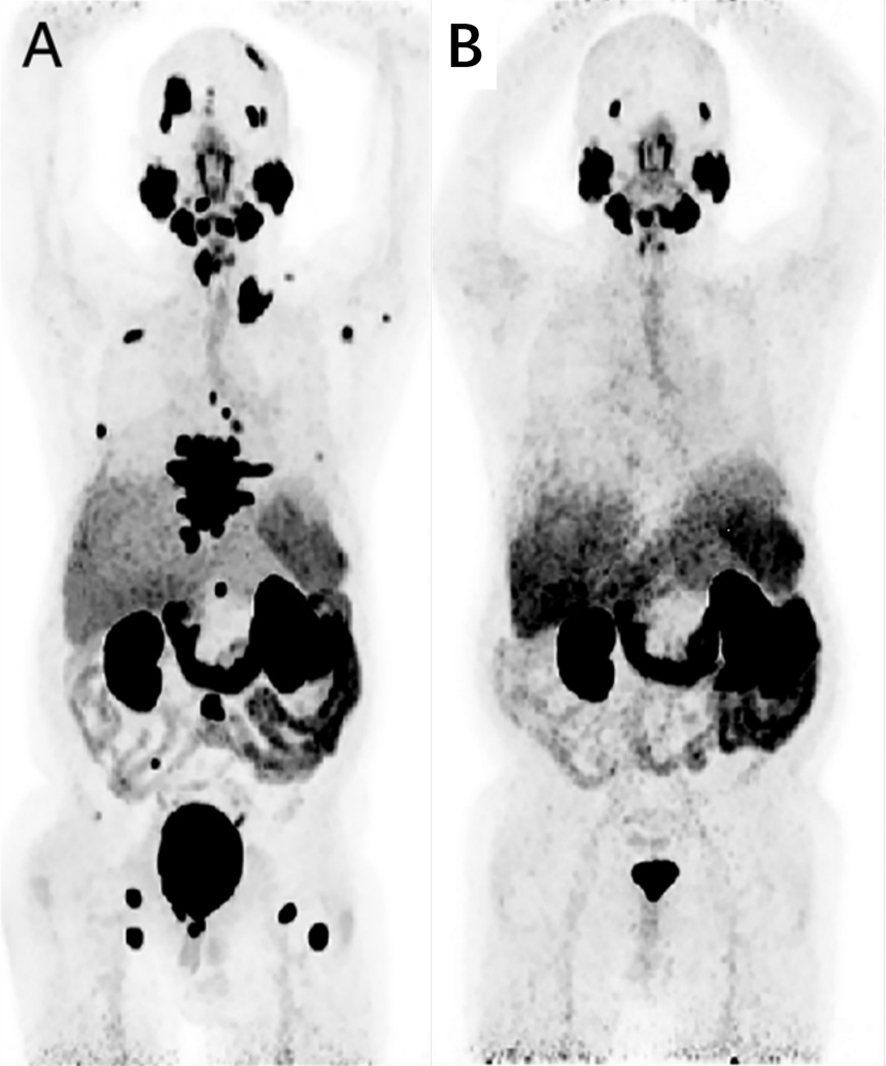
A 65-year-old man, diagnosed with prostate cancer 7 years ago during prostatectomy, experienced biochemical recurrence. A baseline ^68^Ga-PSMA-11 PET/CT imaging was conducted, and the maximum intensity projection (MIP) image revealed systemic PSMA metastases, particularly in bone metastatic lesions **(A)**. Following 2 cycles of Lu-PSMA-617 treatment, a reassessment of treatment efficacy was performed using ^68^Ga-PSMA-11 imaging. The MIP image showed no tracer uptake in new lesions, and there was a significant reduction in tracer uptake overall **(B)**. Adapted from Ref (81).

**Figure 9:**
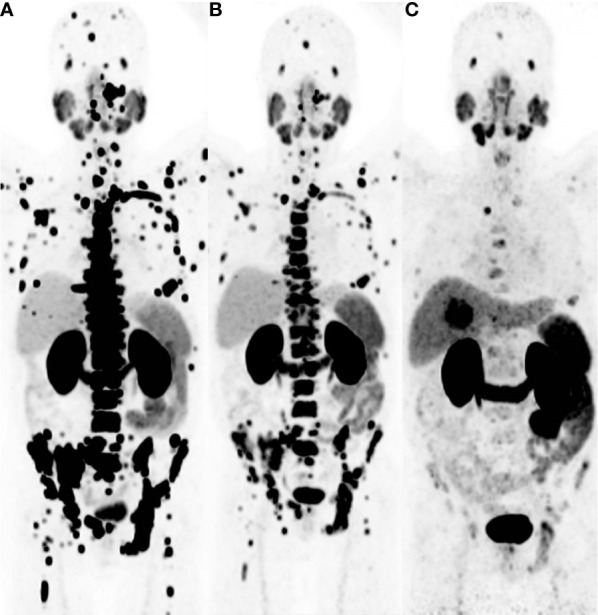
This 63-year-old male patient was diagnosed with prostate cancer 6 years ago and underwent a ^68^Ga-PSMA PET/CT imaging examination. The maximum intensity projection (MIP) image revealed a significant increase in tracer uptake in the skeletal region **(A)**. The patient received 4 cycles of ^225^Ac-PSMA-617 therapy, and after 2 treatment cycles, subsequent ^68^Ga-PSMA PET/CT imaging still showed multiple areas of tracer uptake in the skeletal region, despite a notable reduction in SUVmax **(B)**. A ^68^Ga-PSMA PET/CT imaging performed 16 weeks after the last treatment showed a substantial decrease in tracer uptake in the skeletal lesions **(C)**. Adapted from Ref (82).

The development and application of PSMA ligands in the field of prostate cancer diagnosis and treatment have witnessed remarkable progress in recent years. From the pioneering ^68^Ga-PSMA-11 to the therapeutic breakthroughs with ^177^Lu-PSMA-617 and other emerging compounds, these radiolabeled PSMA-targeting agents have significantly enhanced our ability to detect and manage prostate cancer. The continuous evolution and integration of these agents into clinical practice hold tremendous potential for personalized and effective management of prostate cancer patients in the future. (**Figures 6**–**9**).

## Author contributions

YY: Conceptualization, Writing – review & editing, Writing – original draft. ZH: Writing – review & editing, Visualization. LT: Writing – review & editing, Visualization. ZJ: Writing – review & editing, Visualization. TM: Writing – review & editing, Visualization. CY: Writing – review & editing, Conceptualization.
